# Water deficit enhances the transmission of plant viruses by insect vectors

**DOI:** 10.1371/journal.pone.0174398

**Published:** 2017-05-03

**Authors:** Manuella van Munster, Michel Yvon, Denis Vile, Beatriz Dader, Alberto Fereres, Stéphane Blanc

**Affiliations:** 1 BGPI UMR385, INRA Montpellier, France; 2 LEPSE UMR759, INRA, Montpellier, France; 3 Department de Protección Vegetal, Instituto de Ciencias Agrarias, Madrid, Spain; University of Basel, SWITZERLAND

## Abstract

Drought is a major threat to crop production worldwide and is accentuated by global warming. Plant responses to this abiotic stress involve physiological changes overlapping, at least partially, the defense pathways elicited both by viruses and their herbivore vectors. Recently, a number of theoretical and empirical studies anticipated the influence of climate changes on vector-borne viruses of plants and animals, mainly addressing the effects on the virus itself or on the vector population dynamics, and inferring possible consequences on virus transmission. Here, we directly assess the effect of a severe water deficit on the efficiency of aphid-transmission of the *Cauliflower mosaic virus* (CaMV) or the *Turnip mosaic virus* (TuMV). For both viruses, our results demonstrate that the rate of vector-transmission is significantly increased from water-deprived source plants: CaMV transmission reproducibly increased by 34% and that of TuMV by 100%. In both cases, the enhanced transmission rate could not be explained by a higher virus accumulation, suggesting a more complex drought-induced process that remains to be elucidated. The evidence that infected plants subjected to drought are much better virus sources for insect vectors may have extensive consequences for viral epidemiology, and should be investigated in a wide range of plant-virus-vector systems.

## Introduction

Drought is a major limiting abiotic stress threatening plant species communities and crop production [1], and global warming increases both its frequency and severity [2, 3]. While improving tolerance to drought in crop plants appears as a necessity [4], another challenge is to predict and anticipate how its increased intensity/frequency will interfere with other stresses, and in particular biotic stresses.

Plant viruses are responsible for tremendous agronomic and socio-economic damages [5]. Nearly all plant viruses are transmitted by vectors, which can be fungi, nematodes, mites, and most importantly insects [5–7]. A growing body of data is uncovering the intimate entanglement of the plant physiological pathways involved in responses to various abiotic stresses and in defense against pathogens and herbivores [8, 9]. Therefore, the plant response to water stress may interfere not only with plant-virus but also with plant-vector interactions, ultimately impacting on the spread of the corresponding viral diseases [10, 11]. A number of studies have been published on the influence of climate change on vector-borne diseases of plants and on their spread. Among these studies, those concerning viruses have long focused either on the vector biology (e.g. developmental time, longevity, fecundity, migration) and ecology [12–14], or on the virus accumulation and symptom expression *in planta* [15–17]. While most of these studies speculate on a possible impact of environmental changes on the rate of virus transmission, direct experimental support was totally lacking, or not statistically tested [18], until very recently [19, 20].

In particular, Dader and colleagues [19] have demonstrated that, in conditions of elevated CO_2_, the biomass production of pepper host plants increased, whereas the population growth of the aphid vector *Myzus persicae* and the transmission rate of *Cucumber mosaic virus* (CMV) decreased. Similarly, Chung et al. [20] have shown that virus acquisition of *Potato virus* Y, *Potato virus* A and *Potato leafroll virus*, decreased when temperature was higher than 20°C. Concerning severe drought, it has long been known that viral infection can protect the host plants by delaying irreversible wilting [21]. Interestingly in wheat, this drought-protective effect of *Barley yellow dwarf virus* (BYDV) is accompanied by an increase of the plant quality for the aphid vector *Rhopalosiphum padi* [11]. While this indicates that severely water-stressed BYDV-infected (*versus* healthy) wheat plants survive better and promote better growth of aphid vector populations, the actual rate of BYDV aphid-transmission from these plants has not been experimentally tested.

Here, we directly estimate the effect of severe water deficit on the transmission of 2 unrelated virus species, *Cauliflower mosaic virus* (CaMV, Family *Caulimoviridae*) and *Turnip mosaic virus* (TuMV, Family *Potyviridae*), by the aphid vector *Myzus persicae* in turnip (*Brassica rapa* cv. ‘Just Right’).

## Materials and methods

### Plant material and growth conditions

Turnip (*Brassica rapa* cv. ‘Just Right’) was used both as infected source plants and as young test plantlets for aphid transmission assays (see below). Plants were individually grown in 9 x 9 x 9 cm square pots containing equal amount of substrate (Humin-substrat N2, Neuhaus; Klasmann-Delmann, Geeste, Germany). The field capacity (FC) of the substrate was 0.8 g H_2_O g^−1^ dry soil, as indicated by the manufacturer. Plants were maintained in an insect-free walk-in growth chamber with a photoperiod of 12/12 h (day/night) set at 23/21°C (day/night) with 53% air relative humidity. Soil water content was maintained at 0.8 g H_2_O g^−1^ dry soil with a nutrient solution (N 168 mg l^−1^, P 115mg l^−1^, K 336 mg l^−1^, CaO 162 mg l^−1^, MgO 19 mg l^−1^) until the application of the water deficit treatment (see below).

### Virus isolates and inoculation of source plants

Two totally unrelated virus species transmitted in a non-circulative non-persistent manner were independently analyzed, the CaMV isolate Cabb B-JI [22] and the TuMV-UK1 [23], both efficiently transmitted by the aphid species *M*. *persicae* [24].

To produce the infected plants used as virus source plants in transmission assays, CaMV and TuMV were inoculated in young turnip seedlings at the 2-leaf (L2) developmental stage by mechanical sap inoculation as previously described [25, 26].

### Aphid population

The colony of the aphid-vector species *M*. *persicae* was collected over 30 years ago in the south of France [27]. It is maintained on eggplants (*Solanum melongena*) in insect-proof cages, in a growth chamber at 23/18°C (day/night) with a photoperiod of 14/10 h (day/night), in conditions ensuring clonal reproduction. Aphids were transferred to new cages and to new host plants every 2 weeks, in order to avoid overcrowding and induction of the development of winged morphs.

### Watering deficit treatment of infected source plants

In all experiments, the water deficit (WD) regime was initiated 2 weeks post-inoculation, corresponding to the approximate time of appearance of the first symptoms of systemic infection for both CaMV and TuMV. At this date, watering was first totally stopped for 6 days. At day 6 of the WD regime, the soil water content was partially restored and adjusted to 0.5 ± 0.05 (s.d.) g H_2_O g^−1^ dry soil by adding 50 ml of the nutrient solution per plant pot, corresponding to 66% of FC (S1 Fig). Watering was then held for 3 additional days. At day 9 of the WD regime, 2 hours prior to transmission assays (see below), the soil water content was finally adjusted to 0.4 ± 0.02 (s.d.) g H_2_O g^−1^ dry soil with 50 ml of the nutrient solution, so 50% of FC (see S1 Fig), in order to restore leaf turgor and homogenize the plant palatability for aphid vectors. For control well-watered (WW) plants, the soil water content was constantly maintained at 0.8 ± 0.03 (s.d.) g H_2_O g^−1^ dry soil, corresponding to 100% of FC (S1 Fig), as indicated in the “Plant material and growth conditions” section above.

Two additional experiments using CaMV-infected plants were conducted with the exact same WD and WW regimes but using “clear” water (tap running water non supplemented with nutrient elements) instead of the nutrient solution. These 2 additional experiments were performed in order to control for a putative unwanted effect of nutrient deprivation in the WD treatment compared to WW on efficiency of virus transmission.

### Plant traits estimates

To estimate the plant development and growth under the indicated experimental conditions and at indicated dates, leaf length was measured from the base of the petiole to the tip of the lamina on 3 fully expanded leaves per plant (L4, L6 and L8). The phyllochron, i.e. the average time required for the emergence of a newly formed leaf during the indicated period, was determined by daily visual recording. For estimating the total dry mass of green tissues produced by plants under the 2 watering regimes, leaves of all source plants were collected at the end of transmission assays and placed in an oven 48 h at 65°C in order to determine their dry weight / treatment (WW or WD).

The water potential under transpiring conditions (Ψ), as indicative of minimal daytime values, was measured on the leaf 8 (L8) of each analyzed plant at the end of the water deprivation regime. This analysis was performed in a controlled growth chamber between 3 and 4 hours after lights were switched on, with a Scholander pressure chamber (Soil Moisture Equipment Corp., Santa Barbara, CA, USA) which was cross-calibrated using a distributed, pressurized nitrogen source [28].

### Aphid transmission assays

For transmission experiments, batches of 20 nymphs of *M*. *persicae* (N2-N4 instars) were starved for 1 hour and placed on leaf L8 of an infected source plant. When they stopped walking and inserted their stylets into the leaf surface, they were allowed to feed for a short 2-minutes period. Aphids were then immediately collected in a Petri dish and transferred individually to young 7 days-old seedlings (test plants), for an inoculation period of 3 hours before insecticide spray (1 aphid/test plant; 12 test plants/source plant, and 10 source plants per watering treatment). Symptoms were recorded 3 weeks later by visual inspection to evaluate virus transmission rate. After transmission assays, 3 leaf discs (0.8 cm diameter) distributed evenly over the leaf surface were sampled from L8 of each infected source plant, and stored at -80°C for further nucleic acid extraction and quantification of the virus accumulation.

### Plant DNA and RNA extraction

Total DNA from CaMV-infected leaf disc samples (pools of the 3 leaf discs collected per plant) was extracted according to a modified Edwards protocol [29] with an additional washing step with 70% ethanol. DNA was resuspended in 200 μl of water, and 10-fold dilutions were used as qPCR templates.

Total RNA from TuMV-infected leaf-disc samples (pools as above) was extracted using the RNeasy kit (Qiagen) following manufacturer’s instructions prior to cDNA synthesis.

The quality and quantity of nucleic acid extraction was assessed by spectroscopic measurements at 230, 260 and 280 nm (NanoDrop 2000 Spectrophotometer).

### Single-stranded cDNA synthesis

One microgram of total RNA extracted from TuMV-infected leaf-discs samples was used for single-strand cDNA synthesis with 1.5 μg of specific reverse primer (Tu8907-R: 5’-TTTCCCTCTTCTTGTGCAACATCC-3’, or R-ActBra: 5’-GATCTCTTTGCTCATACGGTCTG-3’) and Avian Myeloblastosis Virus (AMV) reverse transcriptase (Promega kit) according to the manufacturer’s instructions.

Finally, the reverse transcriptase product was diluted to 1/10 prior to qPCR analysis.

### DNA quantification by qPCR

DNA quantification was performed in duplicates by real time quantitative PCR (qPCR) in 384-well optical plates using the LightCycler FastStart DNA Master Plus SYBRgreen I kit (Roche) in a LightCycler 480 thermocycler (Roche), following the manufacturer’s instructions. Specific sets of primers designed for quantification of CaMV (Ca4443-F: 5’-GACCTAAAAGTCATCAAGCCCA-3’ and Ca4557-R: 5’-TAGCTTTGTAGTTGACTACCATACG) and TuMV (Tu8767-F: 5’-AACGCTTGATGCAGGTTTGAC-3’ and Tu8907-R) genomes were used at a final concentration of 0.3 μM. All qPCR reactions were carried out with 40 cycles (95°C for 15 s, 62°C for 15 s and 72°C for 15 s) after an initial step at 95°C for 10 min. The qPCR data were analyzed with the LinReg PCR program to account for the efficiency of every single PCR reactions [30]. The absolute initial viral concentration in *B*. *rapa* plants, expressed in arbitrary fluorescence units: N_0_ CaMV or N_0_ TuMV, was respectively divided by that of a host plant gene or of its mRNA (N_0_ actin; *B*. *napus* actin gene, Genbank accession GQ 339782.1; primers R-ActBra and F-Act2: 5'- GACYTBTAYGGTAACATTGTGCTC-3’), in order to normalize the amount of plant material analyzed in all samples. Actin is a commonly used house-keeping gene and was previously used for quantification of gene expression in plants under water stress [31].

### Statistical analysis

The effects of the water regime (WW vs. WD) on plant traits or on viral accumulation (log (N_0_ virus/N_0_ actin)) were assessed using separate ANOVAs, accounting for the possible ‘block’ effect between experimental replicates (n = 2–3 experiments depending on the response variable; see S1 Table). The significance of the different effects was assessed using the Student *t*-test (*p* ≤ 0.05). Where not significant, the block effect was removed and the model was tested again with pooled data.

The relationship between transmission rate, viral accumulation in source plants and water regime was analyzed using a binomial generalized linear model (GLM) with the conventional logit link function, accounting for a possible “block” effect between experiment replicates (n = 3 for each virus; see S1 Table). The coefficients of the GLM were determined using maximum likelihood estimators, and their CI_95_ was calculated using normal quantiles and standard error. CaMV and TuMV transmission were compared using a χ^2^ test on the likelihood ratios (*p* ≤ 0.05) to check if the observed frequency distribution was related to the expected frequency distribution.

All statistical analyses were carried out with JMP software (JMP 10, SAS Institute Inc.).

## Results

### Effects of water deprivation on the phenotype of infected turnip host plants

**Fig 1:**
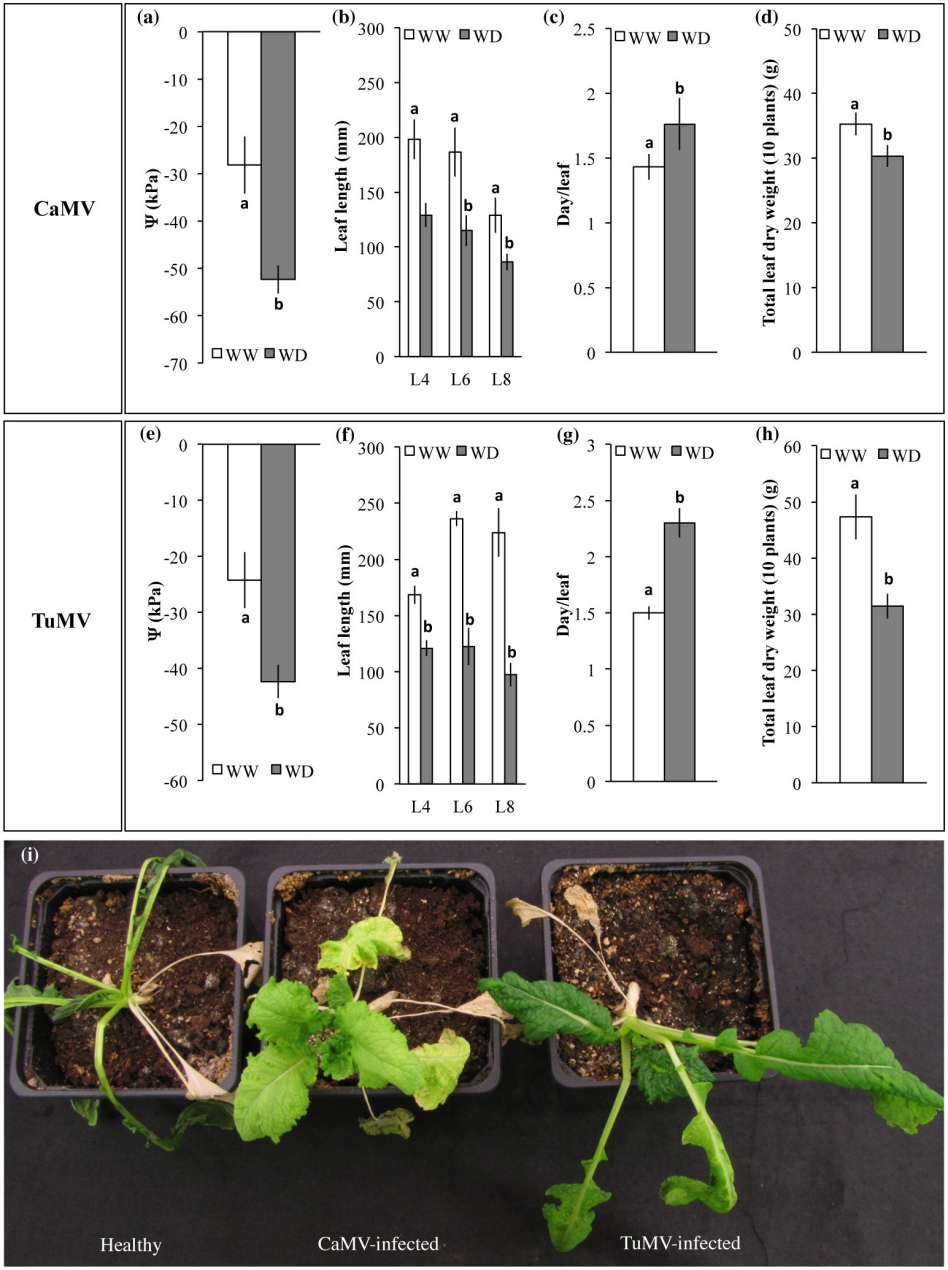
Morpho-physiological traits of CaMV- and TuMV-infected plants grown under Well-Watered (WW) or Water Deficit (WD) conditions. (a, e) Water potential (Ψ) of L8 of CaMV or TuMV infected plants at the end (Day 9) of the water deprivation regime (n = 12 for both CaMV and TuMV infected plants). (b, f) Length of fully expanded leaves L4, L6 and L8 of CaMV or TuMV infected plants (n = 20 per leaf level for both virus species). (c, g) average time required for the appearance of each newly formed leaf on CaMV or TuMV infected plants (n = 20 in both cases). (d, h) Total dry weight of leaves from pools of 10 CaMV or 10 TuMV infected plants (n_pools_ = 3 in both cases). (i) Illustration of the protective effects of CaMV and TuMV infection on turnip response to severe water deficit. Healthy (mock-inoculated), CaMV-infected and TuMV-infected plants are shown 30 days after total water withholding. (a-h) Bars represent SEM and different lowercase letters indicate significant differences between water treatments according to a Student’s *t*-test (*p* ≤ 0.05, see text). Details on plants used in the different morpho-physiological traits measurements are summarized in S1 Table.

The water deprivation regime described in the Methods section reduced the soil water content by 50% (S1 Fig) and had strong effects on plant growth and development (Fig 1). At the end of the treatment, i.e. the day of the aphid-transmission assays, the water potential of the leaves used as virus sources (leaves L8) was significantly reduced down to half that of unstressed control plants infected with CaMV or TuMV (*t*-test, *p*<0.001 for both CaMV and TuMV infected plants; Fig 1a and 1e).

The water-deprived plants infected with either CaMV or TuMV showed a reduction of leaf length (*t*-test, *p<*0.01 and p<0.001, respectively; Fig 1b and 1f), a delay in leaf production (*t*-test, *p<*0.01 and *p*<0.001, respectively; Fig 1c and 1g), and a reduction of aboveground dry mass (*t*-test, *p<*0.01 and *p*<0.001, respectively; Fig 1d and 1h), when compared to well-watered infected plants.

Although not the primary objective of this study and thus not formally quantified, it should be mentioned that the viral infection (both with CaMV and TuMV) somewhat protected the host plants from extreme water deficit, consistent with results earlier reported on several other plant-virus species associations [21]. In experiments where non-infected plants were submitted to an extreme water deprivation regime, the wilting of leaves was more pronounced and appeared earlier in healthy plants. As a consequence, plant death occurred more rapidly in healthy than in infected plants (Fig 1i).

### Water deficit increases the quality of host plants as virus sources for aphid-transmission

**Fig 2:**
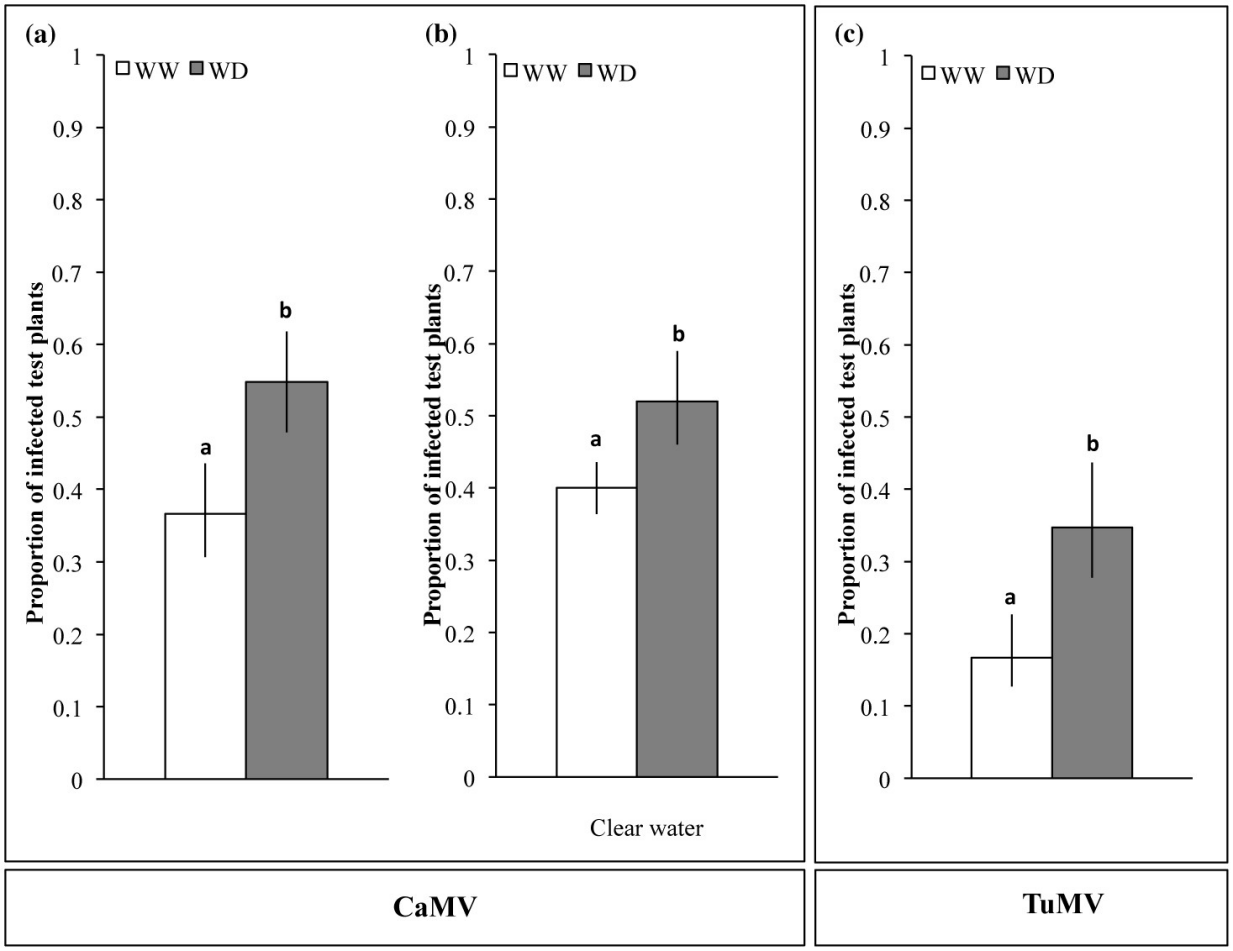
Aphid-transmission of CaMV and TuMV from turnip plants grown under Well-Watered (WW) or Water Deficit (WD) conditions. (a-c) Means of the proportion of infected test plants calculated from a pool of three independent experiments for each viral species (a, c) and two additional experiments conducted with CaMV-infected plants (b). In each experiment and for each condition, n = 10 source plants and n = 12 aphids per source plants individually transferred onto n = 12 receiving test plants. Watering was performed with a nutrient solution (a, c) or clear water (b). Error bars represent 95% confidence intervals and different lowercase letters indicate significant differences between water treatments according to a χ^2^ test on the likelihood ratios (*p* ≤ 0.05) for both CaMV and TuMV.

**Fig 3:**
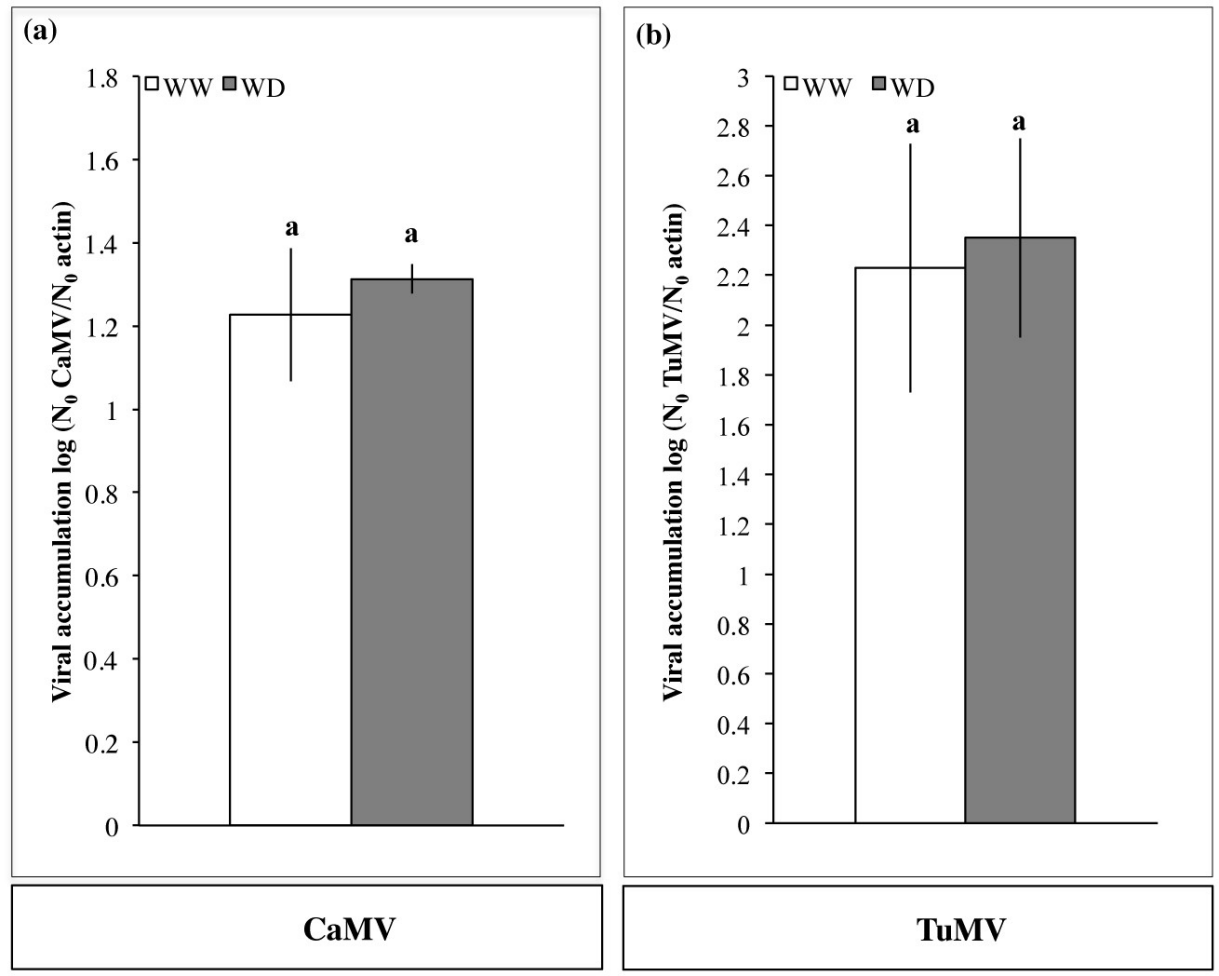
Effect of water deficit on the virus accumulation in infected source plants. Viral accumulation in the infected source plants used in transmission assays was estimated in leaf 8 by qPCR and RT-qPCR for (a) CaMV- and (b) TuMV- infected plants, respectively (n = 30 for both the WW and WD conditions). Bars represent 95% confidence intervals of normalized viral accumulation (log (N_0_ virus/N_0_ actin)), and similar lowercase letters indicate non-significant differences between water treatments according to a Student’s *t*-test (*p* ≤ 0.05) for CaMV and TuMV, respectively.

The water deprivation regime applied to the host plants increased significantly the transmission rate of both CaMV and TuMV in 3 replicate experiments (Fig 2; Table 1). For CaMV-infected plants, small but significant variations between replicates were detected in the GLM analysis (*p* = 0.011), however no significant interaction was found with the watering regime (*p* = 0.832). On average, the transmission of CaMV increased by 34% under WD (26–44% across experiments, not shown; χ^2^ = 30.99, df = 2, *p<*0.001) (Fig 2a). To make sure that the observed transmission increase was due to water shortage rather than reduction of nutrient input in the WD regime, we repeated the exact same experiment with clear water (no nutrient added in either WD or WW regimes, see Methods). In this control, and thus in the total absence of added nutrients, the aphid-transmission of CaMV similarly increased by 30% under WD compared to WW conditions (*p*<0.001, Fig 2b). The virus accumulation expressed as log (N_0_ CaMV/N_0_ actin) did not significantly change in response to WD (*t* = 2.04, df = 53.27, *p* = 0.06; Fig 3a). Consistently, the CaMV transmission rate did not significantly correlate with virus accumulation in the source plants (*p* = 0.232), neither in distinct watering regimes nor in distinct experimental repeats (*p* = 0.637 and 0.324, respectively, Table 1).

**Table 1:** Effect of watering, virus accumulation and experimental replicate on aphid transmission efficiency of CaMV and TuMV.

Response trait	Factors^a^ and interactions	Full model^b^			Reduced model		
		df^c^	Deviance	*P*-value	df^c^	Deviance	*P*-value
Transmission rate of CaMV^a^	Watering	1	**24.56**	<0.0001	2	**30.99**	<0.0001
	Virus accumulation (log)	1	1.42	0.232			
	Replicate	2	**9.93**	0.007	2	**8.986**	0.011
	Watering x Accumulation	1	0.222	0.637			
	Watering x Replicate	2	0.367	0.832			
	Accumulation x Replicate	2	2.252	0.324			
	Replicate x Accumulation x Watering	2	3.253	0.197			
Transmission rate of TuMV^a^	Watering	1	**30.62**	<0.0001	2	**214.6**	<0.0001
	Virus accumulation (log)	1	**18.28**	<0.0001	2	**18.27**	0.0002
	Replicate	2	0.489	0.783			
	Watering x Accumulation	1	0.357	0.550			
	Watering x Replicate	2	1.391	0.500			
	Accumulation x Replicate	2	0.990	0.610			
	Replicate x Accumulation x Watering	2	**10.40**	0.006			

^a^ Factors are watering regime (nutrient solution; well-watered vs. water deficit), virus accumulation (log-transformed) and experimental replicate (n = 3).

^b^ The full model, including all factors and interactions, is tested using a generalized linear model (GLM) with binomial (logit) link function.

^c^ The reduced model includes only significant terms after sequential removal of non-significant terms and interaction.

For TuMV-infected plants, no significant variation in the transmission rate was observed between experimental replicates (*p* = 0.783, Table 1). Overall, the transmission of TuMV was doubled under WD compared to WW conditions (100% increase; χ^2^ = 214.6, df = 2, *p <* 0.001; Fig 2c). For this virus species, we detected a significant correlation between the transmission rate and the viral accumulation in the GLM analysis (*p* < 0.001), but it cannot explain increased transmission from water-deprived source plants. Indeed, accumulation of TuMV in infected plants was not significantly different under WD compared to the WW condition (*t* = 1.50, df = 56.67, *p* = 0.14; Fig 3b).

## Discussion

Insect-vectored plant viruses are near-ubiquitous components of natural and agricultural ecosystems and often reach a very high incidence both in crops and in more complex plant communities [32, 33]. It is anticipated that abiotic stresses can impact multiple steps of the intricate plant-virus-vector interaction and so modify the transmission rates in many different ways [10, 34]. Here we studied the « direct » effect of water deprivation, a major stress encountered by plants in natural field conditions, on virus transmission using 2 phylogenetically unrelated plant viruses. We focused on a simple experimental design where the parameter tested is the impact of drought on infected plants regarding their capacity to act as a virus source for aphid vectors. Neither the aphids nor the receptor test plants were submitted to any particular stress, hence specifically informing on the efficacy of virus acquisition depending on water availability or deficiency to infected plants. In these conditions, we showed that a severe water deficit applied to CaMV- or TuMV-infected plants dramatically enhanced transmission by around 34% and 100%, respectively.

Virus accumulation in source plants was evaluated as a potential explanatory factor, since its positive correlation with the transmission success has been reported in some instances [35, 36], though not in others [37]. For both CaMV and TuMV, the increased transmission from water-stressed infected leaves cannot be attributed to increased viral accumulation because no correlation could be demonstrated between these 2 traits (Table 1). Another possible explanation is that aphids may feed differently on WD infected plants, due to plant quality (e.g. higher sugar contents [38]) as this simple fact could explain different transmission success [39, 40]. In all transmission assays, we scrutinized all aphids one by one when deposited on the source leaf, until they stopped moving, inserted their stylets into superficial tissues, and fed for 2 minutes (see Methods). We did not observe any distinct aphid behavior related to water deficit of the host plant. However, such a visual inspection of the aphid behavior could only reveal major changes and more precise and thorough Electrical Penetration Graph-monitoring (EPG) is required to provide a reliable assessment of this possibility. We emphasize that many other unknown factors may be responsible for the observed increased transmission under water deficit conditions. For example, we previously discussed the fact that the physiological status of the host plant could have a direct effect on the virus ‘behavior’ [41]. We consistently showed that CaMV can “sense” the aphid feeding activity, as well as some abiotic stresses, and immediately and reversibly produce transmissible morphs.

Such rapid viral “reaction” actually predisposes the infected plant to a more efficient virus acquisition and transmission by aphid vectors [41, 42]. This remarkable phenomenon has been designated “transmission activation” [43], it can be triggered by abiotic stresses, and whether it also exist in virus species other than CaMV (like for example here TuMV) is currently being investigated.

Thus far, as already mentioned in the Introduction section, very rare papers have experimentally and directly addressed the impact of an abiotic stress on the efficiency of transmission of a plant virus by an insect or other vector [19, 20], and they targeted CO_2_- and heat-related stresses. In the context of climatic change we here provide direct evidence that infected plants subjected to drought facilitate aphid-transmission of distinct virus species, and this independent of other putative factors such as attraction of vectors and modification of their fitness. Although the underlying mechanisms remain at this stage elusive, the resulting drought effect is quantitatively dramatic with a doubling of TuMV transmission rate, indicating that huge fluctuations of a key component of the virus fitness can be anticipated. Together with the consistent observation that several virus species improve plant survival to extreme drought [21] (and Fig 1i), a situation where infected plants survive better adverse environmental conditions and are better sources for viral transmission is imaginable and should be investigated further. Indeed, a clear interaction between biotic (virus) and abiotic (drought) stresses can be seen as a risk to be accounted for in the modeling of virus epidemiology under scenarios of climate changes, but also, and alternatively, as an opportunity to explore biological agents improving tolerance of their host to environmental changes.

## Supporting information

S1 Fig Dynamics of soil water content during the 9 days of water-deprivation regime. Water-deprivation regime was applied 28 days after germination and 14 days after mechanical infection corresponding to appearance of systemic infection for both CaMV and TuMV, exactly as described in the Methods section. Each point represents the mean ± SEM of 20 gravimetric measurements under well-watered (WW, dotted line; 10 pots with CaMV- and 10 pots with TuMV-infected source plants) and under water-deprivation (WD, plain line; 10 pots with CaMV- and 10 pots with TuMV-infected source plants).
(TIF)

S1 Table Experimental replicates and total number of plants used in the experiments. (DOCX)
