# Supplementary figures and images for: Water deficit enhances the transmission of plant viruses by insect vectors
Source: PLoS One. 2017 May 3;12(5):e0174398. doi: 10.1371/journal.pone.0174398 (PMC5414972; doi:10.1371/journal.pone.0174398)

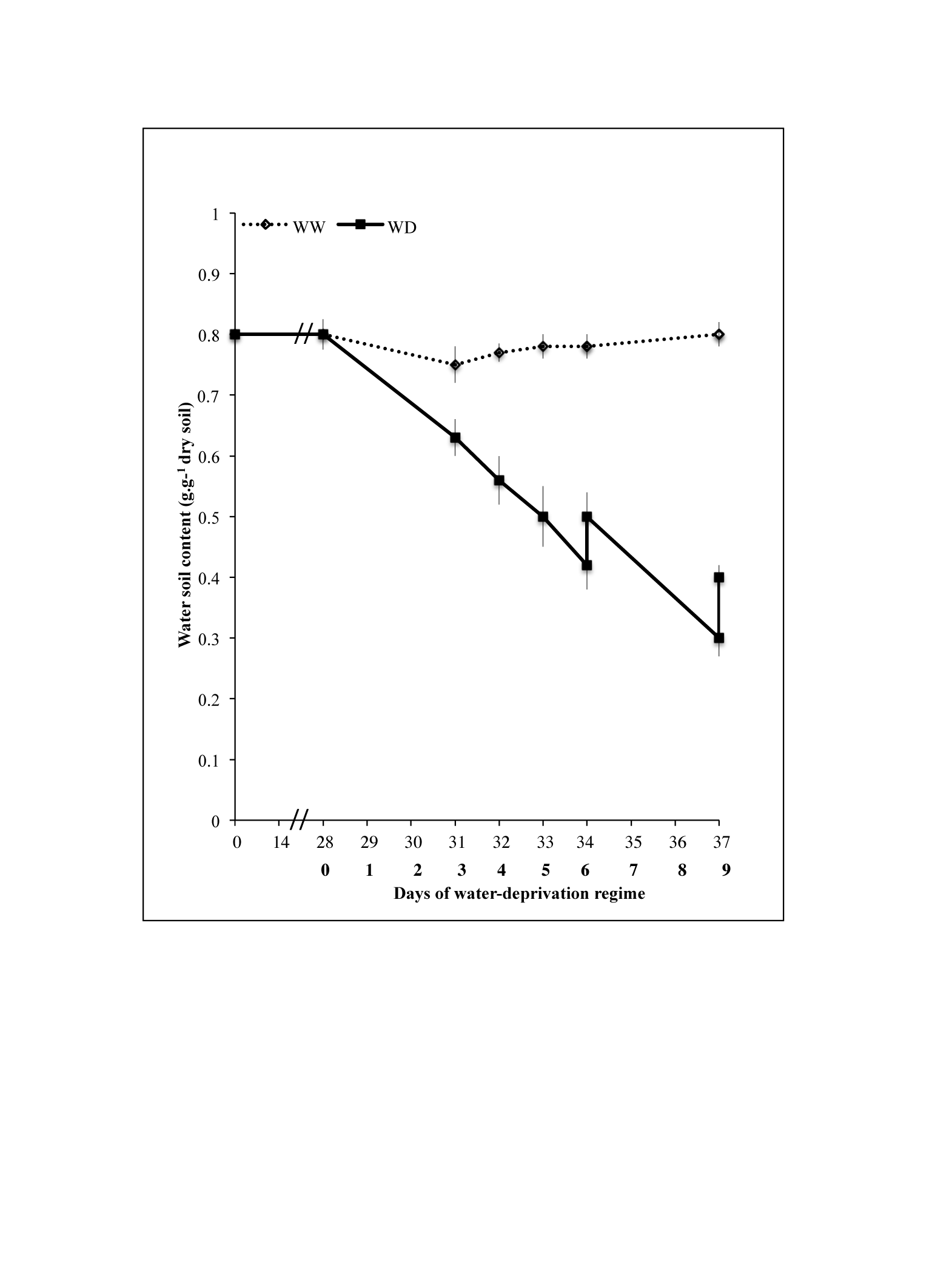

Supplement: S1 Fig — Water-deprivation regime was applied 28 days after germination and 14 days after mechanical infection corresponding to appearance of systemic infection for both CaMV and TuMV, exactly as described in the Methods section. Each point represents the mean ± SEM of 20 gravimetric measurements under well-watered (WW, dotted line; 10 pots with CaMV- and 10 pots with TuMV-infected source plants) and under water-deprivation (WD, plain line; 10 pots with CaMV- and 10 pots with TuMV-infected source plants). (TIF) [file pone.0174398.s001.tif]
