# Supplementary material for: Water deficit enhances the transmission of plant viruses by insect vectors
Source: PLoS One. 2017 May 3;12(5):e0174398. doi: 10.1371/journal.pone.0174398 (PMC5414972; doi:10.1371/journal.pone.0174398)
Supplement: S1 Table — (DOCX) [file pone.0174398.s002.docx]

**S1 Table.** **Experimental replicates and total number of plants used in the experiments**

| Virus | Experiment id | Watering | Watering solution | Leaf length, phyllochron | Total leaf dry mass | Leaf water potential | Transmission assays | | Virus accumulation |
| --- | --- | --- | --- | --- | --- | --- | --- | --- | --- |
|  |  | Source plants | Source plants | Source plants | Source plants | Source plants | Source plants | Test plants ­ | Source plants |
| CaMV | Exp. 1 | WW | NS | 10 | 10 |  | 10 | 120 | 10 |
|  |  | WD | NS | 10 | 10 |  | 10 | 120 | 10 |
|  | Exp. 2 | WW | NS | 10 | 10 |  | 10 | 120 | 10 |
|  |  | WD | NS | 10 | 10 |  | 10 | 120 | 10 |
|  | Exp. 3 | WW | NS |  | 10 |  | 10 | 120 | 10 |
|  |  | WD | NS |  | 10 |  | 10 | 120 | 10 |
|  | Exp. 4 | WW | W |  |  |  | 10 | 120 |  |
|  |  | WD | W |  |  |  | 10 | 120 |  |
|  | Exp. 5 | WW | W |  |  |  | 10 | 120 |  |
|  |  | WD | W |  |  |  | 10 | 120 |  |
|  | Exp. 6 | WW | NS |  |  | 12 |  |  |  |
|  |  | WD | NS |  |  | 12 |  |  |  |
| TuMV | Exp. 1 | WW | NS | 10 | 10 |  | 10 | 120 | 10 |
|  |  | WD | NS | 10 | 10 |  | 10 | 120 | 10 |
|  | Exp. 2 | WW | NS | 10 | 10 |  | 10 | 120 | 10 |
|  |  | WD | NS | 10 | 10 |  | 10 | 120 | 10 |
|  | Exp. 3 | WW | NS |  | 10 |  | 10 | 120 | 10 |
|  |  | WD | NS |  | 10 |  | 10 | 120 | 10 |
|  | Exp. 4 | WW | NS |  |  | 12 |  |  |  |
|  |  | WD | NS |  |  | 12 |  |  |  |

WW: well-watered; WD: water deficit; NS: nutrient solution; W: water. Test plants are 7-days old seedlings grown under well-watered conditions.
